# Tibia‐only planning to 3° valgus implies mechanical medial proximal tibial angle ≥94° in about two‐thirds of varus knee osteoarthritis radiographs

**DOI:** 10.1002/jeo2.70977

**Published:** 2026-11-04

**Authors:** Xu Jiang, Yicang Wang, Xing Li, Songtao Ai, Jun Zhou, Kai Xie, Liao Wang, Mengning Yan

**Affiliations:** ^1^ Department of Orthopaedic Surgery, Shanghai Key Laboratory of Orthopaedic Implants, Shanghai Ninth People's Hospital Shanghai Jiao Tong University School of Medicine Zhizaoju Road Shanghai China; ^2^ Yingwei Medical Technology (Shanghai) Co. Ltd. Shanghai China; ^3^ Department of Radiology, Shanghai Ninth People's Hospital Shanghai Jiao Tong University School of Medicine Shanghai China; ^4^ Institute of Image Communication and Network Engineering Shanghai Jiao Tong University Shanghai China; ^5^ Shanghai Key Laboratory of Digital Media Processing and Transmission Shanghai China

**Keywords:** high tibial osteotomy, joint line convergence angle, knee osteoarthritis, mechanical medial proximal tibial angle, preoperative planning

## Abstract

**Purpose:**

To estimate the proportion of radiographic records from patients with varus knee osteoarthritis in which tibia‐only planning targeting a hip–knee–ankle angle (HKA) of 183° (3° valgus) yielded a calculated mechanical medial proximal tibial angle (mMPTA) of at least 94°.

**Methods:**

In a retrospective database, 1426 knee‐specific records were screened. Eligible records came from adults with standardised standing full‐length lower‐limb radiographs. Exclusions covered hip and foot deformities, spontaneous femoral condyle osteonecrosis, previous lower‐limb surgery, malignancy, major trauma, poor image quality and excessive rotation. Symptomatic knee osteoarthritis, numerical quality control and HKA below 176.5° defined the analytical cohort. Alignment was measured using an artificial intelligence‐assisted system. Correction was assigned entirely to the tibia. The mechanical lateral distal femoral angle and joint line convergence angle were held constant. The primary target was 3° valgus; 1°, 2°, 4°, 5° and 6° of valgus were supplementary targets. Analyses were descriptive, with two‐sided 95% Wilson confidence intervals for proportions.

**Results:**

The analysis included 627 records from 392 patients (mean age, 67.3 ± 9.2 years; 28.6% men). At the 3° valgus target, the mean calculated mMPTA was 96.0 ± 3.7°; 424/627 records reached at least 94° (67.6%; 95% confidence interval, 63.9%–71.2%). The most frequent deformity‐source patterns were intraarticular alone (24.7%) and tibial‐side plus intraarticular (24.4%).

**Conclusions:**

At a 3° valgus target, tibia‐only planning implied an mMPTA of at least 94° in about two‐thirds (67.6%) of records from this varus knee osteoarthritis cohort. Preoperative planning should therefore assess local tibial joint‐line orientation and deformity source alongside the whole‐limb alignment target.

**Level of Evidence:**

Level IV, retrospective radiographic geometry study.

## Graphical abstract

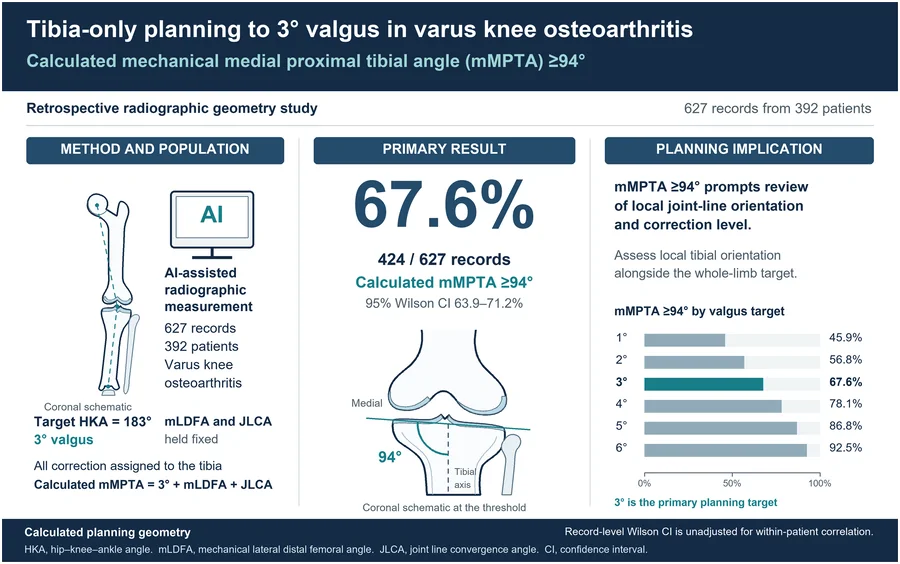

**Abbreviations**
aHKA: arithmetic hip‐knee‐ankle angle
CI: confidence interval
ESSKA: European Society of Sports Traumatology Knee Surgery and Arthroscopy
HKA: hip‐knee‐ankle angle
HTO: high tibial osteotomy
ICC: intraclass correlation coefficient
IQR: interquartile range
JLCA: joint line convergence angle
JLO: joint‐line obliquity
KOA: knee osteoarthritis
K‐L: Kellgren–Lawrence
mLDFA: mechanical lateral distal femoral angle
mMPTA: mechanical medial proximal tibial angle
OWHTO: open‐wedge high tibial osteotomy
QC: quality control
SD: standard deviation
WBL: weight‐bearing line

## INTRODUCTION

Knee osteoarthritis (KOA) is a leading cause of chronic pain, activity limitation and disability in older adults. The Global Burden of Disease 2021 analysis estimated that 595 million people worldwide had osteoarthritis in 2020, representing 7.6% of the global population. The knee was the most commonly affected site. The number of people living with osteoarthritis is projected to increase further by 2050 [28]. Varus alignment is associated with medial compartment loading and radiographic KOA progression [4, 27].

In selected patients with varus KOA, open‐wedge high tibial osteotomy (OWHTO) is used to alter coronal alignment and reduce loading in the medial compartment. Planning usually begins with a whole‐limb hip–knee–ankle angle (HKA) or weight‐bearing‐line target. Previous methods have commonly used approximately 3°–6° of valgus, with the final correction individualised to the patient [7, 10, 15, 22].

Achieving a whole‐limb target through the tibia can substantially alter the local orientation of the proximal tibial joint line. Contemporary planning, therefore, evaluates the mechanical medial proximal tibial angle (mMPTA) and joint‐line obliquity (JLO) together with HKA [5, 24]. Finite‐element analysis has shown that greater joint‐line inclination increases modelled cartilage shear stress [23], providing a biomechanical reason to assess joint‐line orientation during planning. The European Society of Sports Traumatology, Knee Surgery and Arthroscopy (ESSKA) notes that double‐level osteotomy may be considered if a single‐level plan yields mMPTA above 94° or JLO above 5° [5]. A calculated mMPTA of at least 94° was prespecified for planning review.

Previous radiographic simulations showed that the feasibility of isolated tibial correction to 2° valgus depended on the chosen upper limit for mMPTA (90° or 95°) [6]. For planning in symptomatic varus KOA, the relevant questions are how often different valgus targets would require an mMPTA at or above the planning‐review threshold and how femoral alignment and intraarticular deformity contribute to the required tibial correction. Describing these relationships can identify plans in which local joint‐line orientation and the source of deformity warrant closer assessment before the level of correction is selected.

When the full correction is assigned to the tibia, femoral and intraarticular varus contributions must also be accommodated by the planned change in proximal tibial orientation. The calculated mMPTA, therefore, depends on both these components and the selected valgus target. The primary aim was to estimate the proportion of radiographic records with a calculated mMPTA of at least 94° at an HKA target of 3° valgus. Secondary aims were to describe deformity‐source patterns and the distribution of the sum of the mechanical lateral distal femoral angle (mLDFA) and joint line convergence angle (JLCA). Targets of 1, 2, 4, 5 and 6° valgus were examined as supplementary scenarios.

## MATERIALS AND METHODS

### Study design and data source

The institutional review board of the participating hospital approved this retrospective radiographic study and preoperative planning calculation. The study followed the 2013 Declaration of Helsinki [30]. Written informed consent was waived because the dataset was deidentified before analysis.

Standing full‐length bilateral lower‐limb radiographs were obtained from December 2020 through December 2023 with a Multitom Rax system (Siemens Healthineers). During image acquisition, participants stood with both patellae facing forward.

Full‐length imaging was clinically indicated for suspected or confirmed malalignment, femoral or tibial bony deformity, structural limb‐length inequality or assessment of the mechanical axis, joint‐line orientation or deformity source. The source imaging service supported preoperative planning for peri‐knee osteotomy, knee arthroplasty, limb lengthening and deformity correction, as well as necessary postoperative assessment. The analytical cohort was defined by the study exclusions and varus‐KOA criteria.

### Participants

The source population comprised adults referred for clinically indicated standing full‐length lower‐limb radiography. Eligible records came from adults with standardised standing full‐length lower‐limb radiographs. The exclusion criteria were: (1) hip deformity, including developmental dysplasia of the hip, hip osteoarthritis or femoral head osteonecrosis; (2) foot deformity, such as clubfoot, or spontaneous osteonecrosis of the femoral condyle; (3) a history of lower‐limb surgery, malignant tumour or major trauma; (4) poor image quality, including blurred radiographs, insufficient landmark visibility (including obscuration by excessive overlying adipose tissue in patients with large body habitus) or poor image stitching and (5) excessive lower‐limb rotation. After these criteria were applied, 1426 knee‐specific radiographic records remained. The KOA group comprised patients who presented with knee symptoms and received a diagnosis of KOA from an orthopaedic physician. Body mass index was unavailable and was not used as a selection criterion. Kellgren–Lawrence (KL) grade was used to describe osteoarthritis severity, not to determine eligibility.

HKA was defined as the medial angle between the femoral and tibial mechanical axes; 180° denoted neutral alignment, with lower values denoting varus and higher values denoting valgus [14, 15, 25]. A conventional neutral range is 180° ± 3° [5, 16, 25]. The study operationally defined varus alignment as HKA <176.5°, a stricter cutoff than the 177° lower boundary of that range. The radiographic record was the primary analytical unit, and eligible bilateral and repeated records were retained in the primary descriptive analysis. A supplementary analysis retained one record per patient, selecting the record with the lower HKA when more than one was eligible; this was a descriptive check of the unit‐of‐analysis choice.

### Radiographic measurements

The principal coronal parameters were HKA, mMPTA, mLDFA, JLCA and arithmetic HKA (aHKA = mMPTA – mLDFA). The mMPTA describes the coronal orientation of the proximal tibial joint line relative to the tibial mechanical axis. The mLDFA describes the distal femoral joint line relative to the femoral mechanical axis. The JLCA is the angle between the distal femoral and proximal tibial joint lines. Definitions followed established mechanical‐axis conventions and the published edge‐based measurement framework used for this dataset [15, 16, 25].

The measured HKA, mMPTA, mLDFA and JLCA values were assessed against numerical quality‐control (QC) criteria for analysis. These checks were distinct from the clinical and image‐related exclusion criteria described above. Prespecified acceptable ranges were 75°–105° for mLDFA, 70°–105° for mMPTA, −15° to 15° for JLCA, 140°–220° for HKA and 18–100 years for age. Formula consistency was assessed using the absolute residual of HKA – [mMPTA + (180° – mLDFA) – JLCA]; values greater than 0.01° were flagged.

Lower‐limb alignment was measured with Skyward (Yingwei Medical Technology [Shanghai] Co. Ltd., Version 1.1.2.7), an artificial intelligence‐assisted radiographic measurement system. External validation reported absolute errors of 0.07° to 0.45° for knee‐phenotype‐related parameters and low rates of clinically significant failure [16]. Comparisons of manual and semi‐automatic high tibial osteotomy (HTO) planning tools have also shown the importance of checking outlying measurements [19]. Skyward was used for radiographic measurement; it neither selected an osteotomy nor determined clinical eligibility. The planning calculation did not use proprietary surgical software.

### Deformity‐source classification

Deformity‐source patterns were classified using conventional Paley‐style normal ranges: 85°–90° for mMPTA, 85°–90° for mLDFA and 0°–2° for JLCA [5, 16, 21, 25]. Values beyond the varus‐side boundary defined tibial‐side varus (mMPTA < 85°), femoral‐side varus (mLDFA > 90°) and an intraarticular varus component (JLCA > 2°). Each record was assigned to one of eight mutually exclusive combinations. These categories were used to describe the distribution of deformity sources in the cohort.

For sensitivity analysis, Chinese sex‐specific ±2 standard deviation (SD) reference thresholds from a previous radiographic study of coronal lower‐limb morphology in a Chinese non‐KOA population were applied [14]. This analysis tested how the choice of threshold changed category frequencies; the conventional normal‐range classification remained the primary description.

### Tibia‐only planning geometry and calculated mMPTA

The primary scenario used an HKA target of 3° valgus (HKA 183°), the lower bound of the conventional valgus target range reported in previous OWHTO planning studies [7, 10, 15, 22]. Targets of 1°, 2°, 4°, 5° and 6° valgus were supplementary. The 1° and 2° scenarios illustrated how targets closer to neutral changed the calculated local tibial angle.

The calculation assigned the selected HKA correction entirely to the proximal tibia. The mLDFA and JLCA were held fixed. For a target of V° of valgus, calculated mMPTA = preoperative mMPTA + [(180° + V) – preoperative HKA]. Using HKA = mMPTA + (180° – mLDFA) – JLCA, this simplifies exactly to calculated mMPTA = V + mLDFA + JLCA. At the primary 3° valgus target, a calculated mMPTA of at least 94° therefore corresponds to mLDFA + JLCA of at least 91°. This was a deterministic planning calculation, not a fitted prediction or a measured postoperative outcome.

### Outcomes

The primary outcome was the proportion of records with a calculated mMPTA of at least 94° at the 3° valgus target. This inclusive 94° cutoff was prespecified for descriptive planning review, informed by guidance concerning planned mMPTA above 94° [5, 24], and did not define clinical failure. Secondary outcomes were deformity‐source patterns and the observed distribution of mLDFA + JLCA. Calculated mMPTA at 1°, 2°, 4°, 5° and 6° valgus was supplementary.

K‐L grades were retained as descriptive cohort characteristics [17] and were not used to determine eligibility or calculate mMPTA. For reproducibility assessment, two orthopaedic surgeons independently regraded one randomly selected knee from each of 40 patients on two occasions separated by 2 weeks. General K‐L grading criteria were reviewed before the second session; study cases were not discussed and previous scores were not consulted. The readers remained blinded to each other's ratings and to their own previous ratings. Because K‐L grade is ordinal, quadratic‐weighted Cohen kappa was the primary agreement measure. Supplementary measures were absolute‐agreement, two‐way random‐effects, single‐measure intraclass correlation coefficients (ICC[A,1]), exact agreement and within‐one‐grade agreement.

### Statistical analysis

Continuous variables are reported as mean ± SD or median and interquartile range; categorical variables are reported as counts and percentages. The primary effect‐size measure was the number and proportion of records meeting the prespecified 94° planning‐review threshold. Two‐sided 95% confidence intervals (CIs) for proportions were calculated using the Wilson score method without continuity correction. Main‐cohort intervals used the radiographic record as the sampling unit; supplementary one‐record‐per‐patient intervals used the retained patient‐level records. These intervals do not account for within‐patient correlation. Calculated mMPTA is an exact transformation of measured mLDFA, JLCA and the selected target. No odds ratios, regression models, *p* values or significance threshold were therefore used to relate the calculated result to its own components. All eligible records were included, and no hypothesis‐testing sample‐size calculation or post hoc power calculation was performed. The analyses are descriptive and do not estimate causal, diagnostic or procedure‐selection effects.

The original descriptive analyses were performed with Python 3.12, pandas 2.2.3 and NumPy 2.3.5. The additional Wilson intervals were calculated from the reported aggregate counts using Python 3.12.

## RESULTS

### Study population and sample flow

Of 1426 knee‐specific radiographic records, 1420/1426 met the predefined angular ranges and 1423/1426 met the formula‐consistency tolerance. Among 979 records labelled as KOA, 972 passed all QC checks. Applying HKA < 176.5° left 627 eligible records from 392 patients (Figure 1).

**Figure 1:**
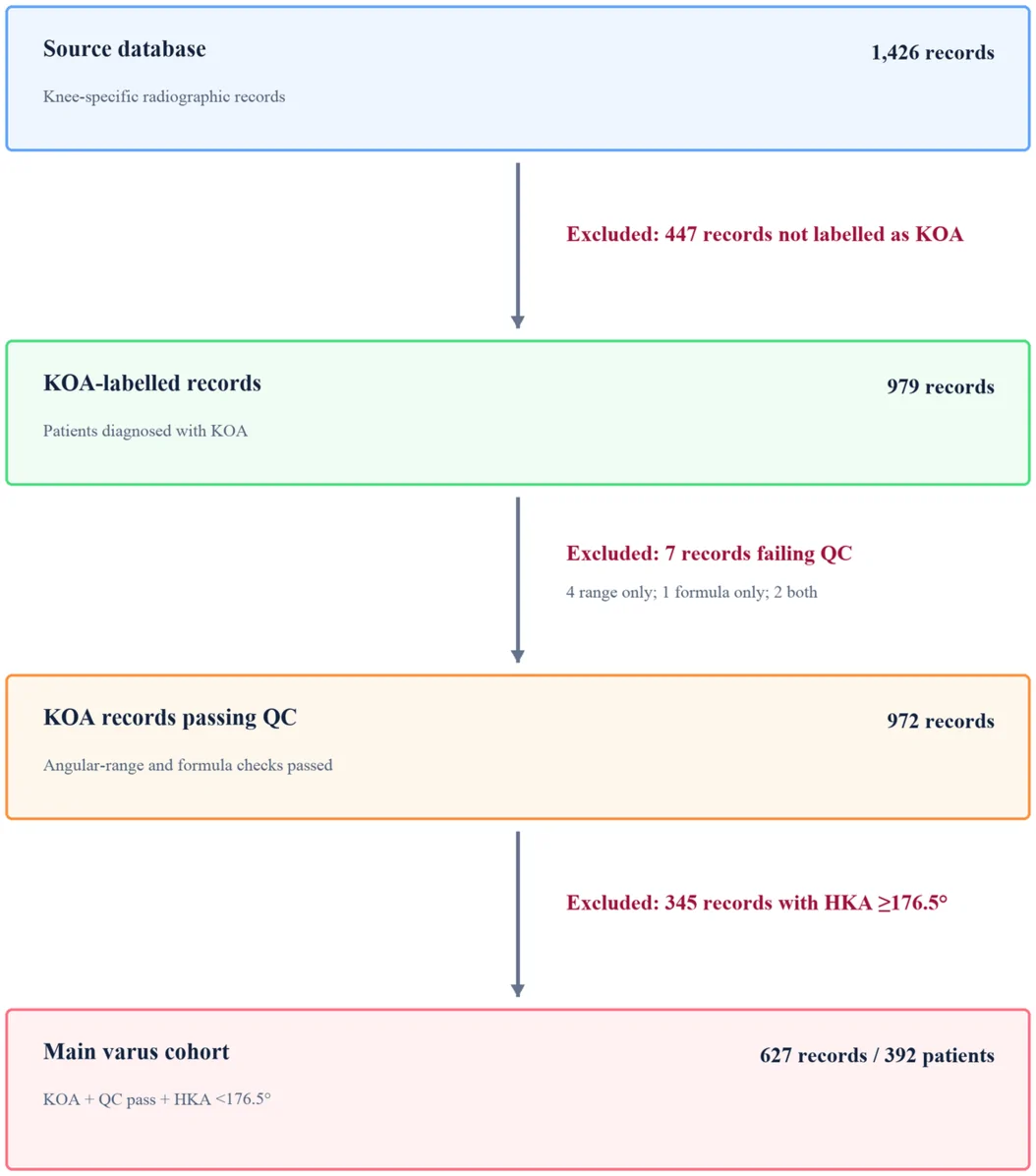
Study flow and analytical cohort. Of 1426 knee‐specific radiographic records, 447 were not labelled as knee osteoarthritis (KOA), seven of 979 KOA‐labelled records failed quality control (QC) and 345 of 972 QC‐passed KOA records had hip‐knee‐ankle angle (HKA) ≥ 176.5°. The final cohort comprised 627 records from 392 patients. QC required age 18–100 years; mechanical lateral distal femoral angle (mLDFA) 75°–105°; mechanical medial proximal tibial angle (mMPTA) 70°–105°; joint line convergence angle (JLCA) −15° to 15°; HKA 140°–220° and an absolute residual ≤0.01° for HKA = mMPTA + (180° – mLDFA) – JLCA.

The 392 patients had a mean age of 67.3 ± 9.2 years (median, 68.0 years; interquartile range, 63.0–73.0 years); 112 (28.6%) were men and 280 (71.4%) were women. Ninety‐eight patients contributed left‐side records only, 79 contributed right‐side records only and 215 contributed bilateral records. The 627 records comprised 322 left‐side and 305 right‐side radiographs. K‐L grades 1, 2, 3 and 4 accounted for 6 (1.0%), 24 (3.8%), 297 (47.4%) and 300 (47.8%) of records, respectively. Body mass index was unavailable in the source export.

Mean preoperative HKA was 171.6° ± 4.0°, corresponding to 8.4° ± 4.0° of varus from neutral. Mean mMPTA, mLDFA, JLCA and aHKA were 84.6° ± 2.9°, 89.1° ± 2.7°, 3.8° ± 2.9° and −4.5° ± 3.5°, respectively (Table 1).

**Table 1:** Characteristics of the main varus knee osteoarthritis cohort.

Variable	Result
Knee‐specific radiographic records analysed	627
Patients	392
Age, mean ± SD, years	67.3 ± 9.2
Age, median (IQR), years	68.0 (63.0, 73.0)
Men	112 (28.6%)
Women	280 (71.4%)
Body mass index	Unavailable in source export
Patients contributing left‐side records only	98 (25.0%)
Patients contributing right‐side records only	79 (20.2%)
Patients contributing bilateral records	215 (54.8%)
Left‐side radiographic records	322 (51.4%)
Right‐side radiographic records	305 (48.6%)
Patient‐side combinations with two eligible radiographs	20
K‐L grade 1	6 (1.0%)
K‐L grade 2	24 (3.8%)
K‐L grade 3	297 (47.4%)
K‐L grade 4	300 (47.8%)
HKA, mean ± SD,°	171.6 ± 4.0
Varus from 180°, mean ± SD	8.4 ± 4.0
mMPTA, mean ± SD,°	84.6 ± 2.9
mLDFA, mean ± SD,°	89.1 ± 2.7
JLCA, mean ± SD,°	3.8 ± 2.9
aHKA = mMPTA—mLDFA, mean ± SD,°	−4.5 ± 3.5

*Note*: Patient‐level age, sex and laterality‐pattern values use 392 patients as the denominator; radiographic measurements, side and K‐L grade use 627 records. Values are presented as *n* (%) unless otherwise indicated. Abbreviations: aHKA, arithmetic hip‐knee‐ankle angle; HKA, hip‐knee‐ankle angle; IQR, interquartile range; JLCA, joint line convergence angle; K‐L, Kellgren–Lawrence; KOA, knee osteoarthritis; mLDFA, mechanical lateral distal femoral angle; mMPTA, mechanical medial proximal tibial angle; SD, standard deviation.

### Deformity‐source distribution

All eight deformity‐source patterns were represented. The intraarticular pattern was present in 155 records (24.7%), and tibial‐side + intraarticular in 153 records (24.4%). The tibial‐side pattern was present in 85 records (13.6%), and femoral‐side + intraarticular in 76 records (12.1%). The tibial‐side + femoral‐side + intraarticular pattern was present in 60 records (9.6%), and tibial‐side + femoral‐side in 40 records (6.4%). The femoral‐side pattern was present in 36 records (5.7%). There was no threshold‐defined abnormality in 22 records (3.5%) (Table 2 and Figure 2).

**Table 2:** Mutually exclusive deformity‐source patterns using conventional normal‐range boundaries.

Deformity‐source pattern	Records	Proportion (%)
Intraarticular	155	24.7
Tibial‐side + intraarticular	153	24.4
Tibial‐side	85	13.6
Femoral‐side + intraarticular	76	12.1
Tibial‐side + femoral‐side + intraarticular	60	9.6
Tibial‐side + femoral‐side	40	6.4
Femoral‐side	36	5.7
No threshold‐defined abnormality	22	3.5

*Note*: Conventional normal ranges were 85°–90° for mMPTA, 85°–90° for mLDFA and 0°–2° for JLCA. Varus‐side abnormalities were defined as mMPTA < 85°, mLDFA > 90° and JLCA > 2°, respectively. Records and proportions are reported as n and %, respectively. Percentages may not total 100% because of rounding.

Abbreviations: JLCA, joint line convergence angle; mLDFA, mechanical lateral distal femoral angle; mMPTA, mechanical medial proximal tibial angle.

**Figure 2:**
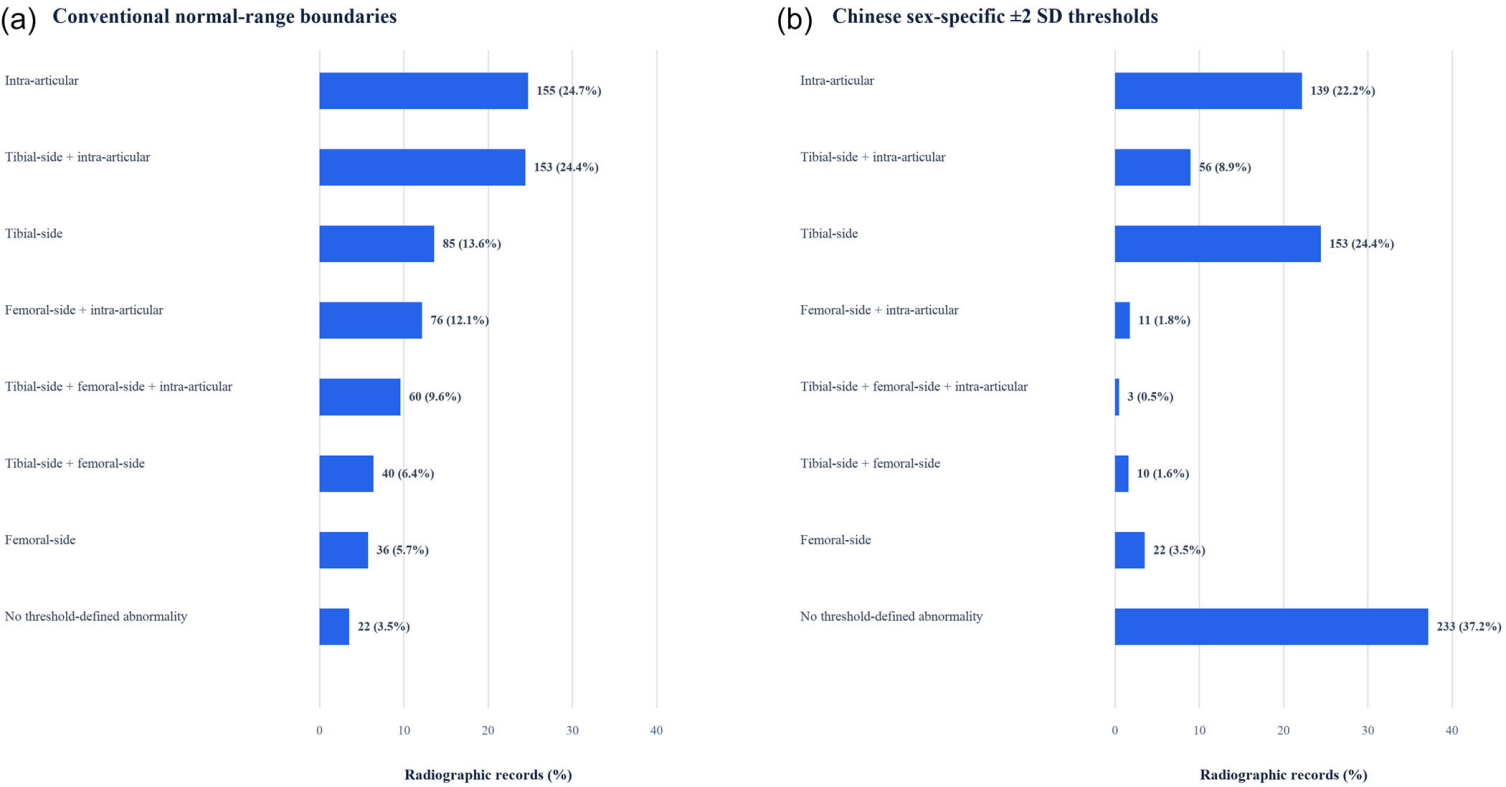
Distribution of coronal deformity‐source patterns in varus knee osteoarthritis (KOA). The eight mutually exclusive patterns were classified using mechanical medial proximal tibial angle (mMPTA), mechanical lateral distal femoral angle (mLDFA) and joint line convergence angle (JLCA). Panel a uses conventional normal‐range boundaries (mMPTA < 85°, mLDFA > 90° and JLCA > 2° for the corresponding varus components); Panel b presents the sensitivity analysis using Chinese sex‐specific mean ±2 standard deviation (SD) thresholds. These classifications describe the anatomical distribution of coronal deformity and do not independently determine the indication for a specific osteotomy procedure.

When Chinese sex‐specific ±2 SD thresholds were applied, category frequencies changed. The largest difference was in the no‐threshold‐defined‐abnormality category, which increased to 233/627 records (37.2%) (Supporting Information: Table S1).

### mMPTA calculated for the selected HKA target

The measured mLDFA + JLCA sum was 93.0° ± 3.7° (range, 84.9°–109.4°). At the primary 3° valgus target, the mean calculated mMPTA was 96.0° ± 3.7°. A calculated mMPTA of at least 94° was found in 424/627 (67.6%; 95% CI, 63.9%–71.2%) of radiographic records. A value above 95° was found in 356/627 (56.8%; 95% CI, 52.9%–60.6%) of records (Table 3 and Figure 3).

**Table 3:** mMPTA calculated for isolated tibial correction at the primary 3° HKA valgus target.

Target HKA	Target HKA value	Calculated mMPTA, mean ± SD	mMPTA ≥ 94°, *n* (%; 95% CI)	mMPTA >95°, *n* (%; 95% CI)
3° valgus	183°	96.0 ± 3.7°	424/627 (67.6%; 95% CI, 63.9%–71.2%)	356/627 (56.8%; 95% CI, 52.9%–60.6%)

*Note*: Values are reported as mean ± SD or *n* (%; 95% Wilson CI). The CIs do not account for within‐patient correlation. The ≥94° endpoint was prespecified; the >95° summary is descriptive. These are planning‐review values rather than clinical failure definitions. The table reports the primary 3° valgus scenario.

Abbreviations: CI, confidence interval; HKA, hip‐knee‐ankle angle; mMPTA, mechanical medial proximal tibial angle; SD, standard deviation.

**Figure 3:**
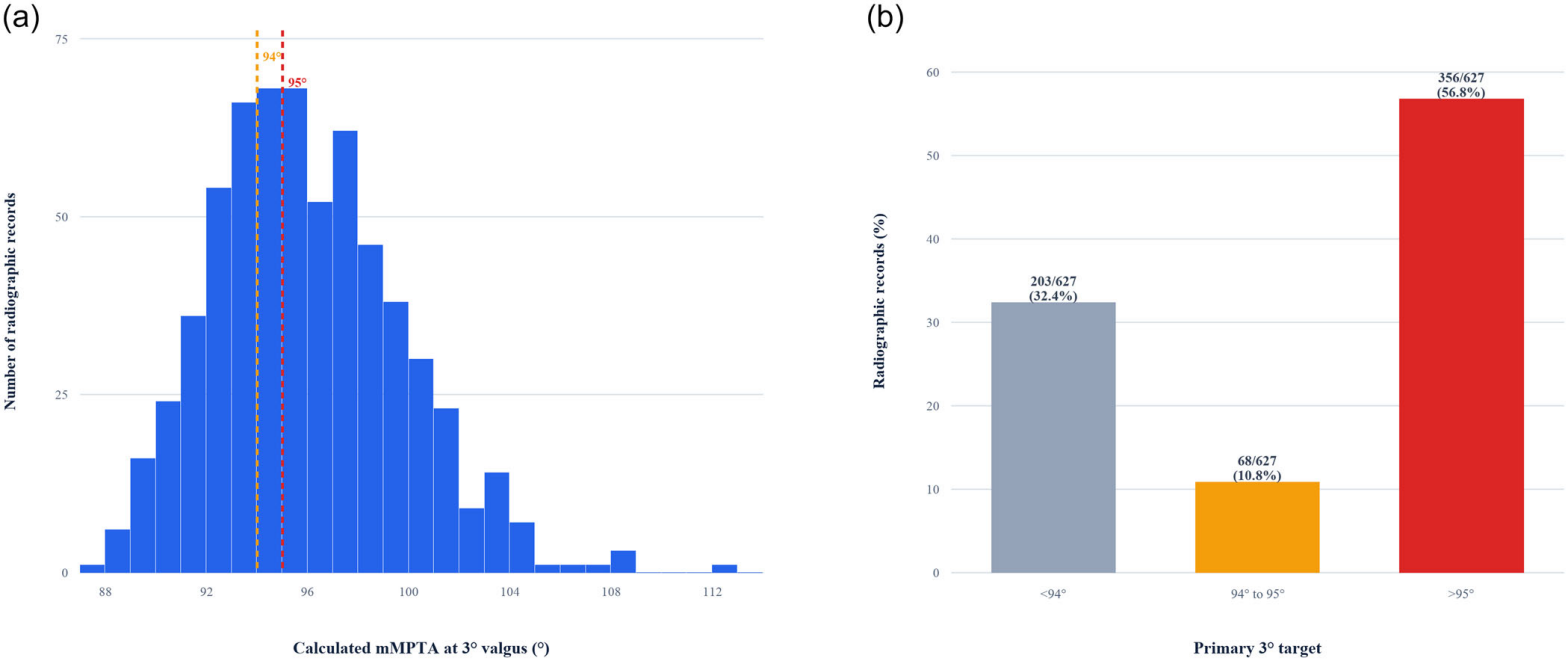
Mechanical medial proximal tibial angle (mMPTA) calculated for isolated tibial correction to the primary target of 3° valgus. The deterministic calculation assumed that the entire change in hip‐knee‐ankle angle (HKA) was produced through the tibia while mechanical lateral distal femoral angle (mLDFA) and joint line convergence angle (JLCA) remained unchanged. Under these assumptions, mMPTA was calculated as 3° + mLDFA + JLCA. (a) Distribution of calculated mechanical medial proximal tibial angle (mMPTA) at the primary 3° valgus hip‐knee‐ankle angle target. (b) Proportions of radiographic records with calculated mMPTA <94°, 94°–95°, and >95°. The thresholds are planning‐review values rather than definitions of clinical failure, and the calculated values are not achieved postoperative measurements. The primary ≥94° proportion was 424/627 (67.6%; 95% Wilson CI, 63.9%–71.2%). This interval does not account for within‐patient correlation. CI, confidence interval.

In the supplementary scenarios, the proportions at or above 94° were 288/627 (45.9%; 95% CI, 42.1%–49.8%) at 1° valgus and 356/627 (56.8%; 95% CI, 52.9%–60.6%) at 2° valgus. The proportion was 490/627 (78.1%; 95% CI, 74.7%–81.2%) at 4° valgus. It was 544/627 (86.8%; 95% CI, 83.9%–89.2%) at 5° valgus and 580/627 (92.5%; 95% CI, 90.2%–94.3%) at 6° valgus (Supporting Information: Table S4). Increasing the target by 1° shifted the entire distribution of calculated mMPTA by 1°.

### Observed geometry underlying the 3° scenario

At the 3° target, the 94° review threshold corresponds exactly to mLDFA + JLCA of 91°. The observed component sum was below 90° in 137 records (21.9%), 90 to less than 91° in 66 records (10.5%), 91 to less than 92° in 68 records (10.8%) and 92° or greater in 356 records (56.8%) (Table 4 and Figure 4).

**Table 4:** Observed mLDFA + JLCA distribution and direct implication for mMPTA at a 3° HKA valgus target.

Observed mLDFA + JLCA	Calculated mMPTA at 3° valgus	Records	Proportion (%)
<90°	<93°	137	21.9
90 to <91°	93 to <94°	66	10.5
91 to <92°	94 to <95°	68	10.8
≥92°	≥95°	356	56.8

*Note*: At a 3° HKA valgus target, calculated mMPTA = 3 + mLDFA + JLCA. Records and proportions are reported as *n* and %, respectively.

Abbreviations: HKA, hip‐knee‐ankle angle; JLCA, joint line convergence angle; mLDFA, mechanical lateral distal femoral angle; mMPTA, mechanical medial proximal tibial angle.

**Figure 4:**
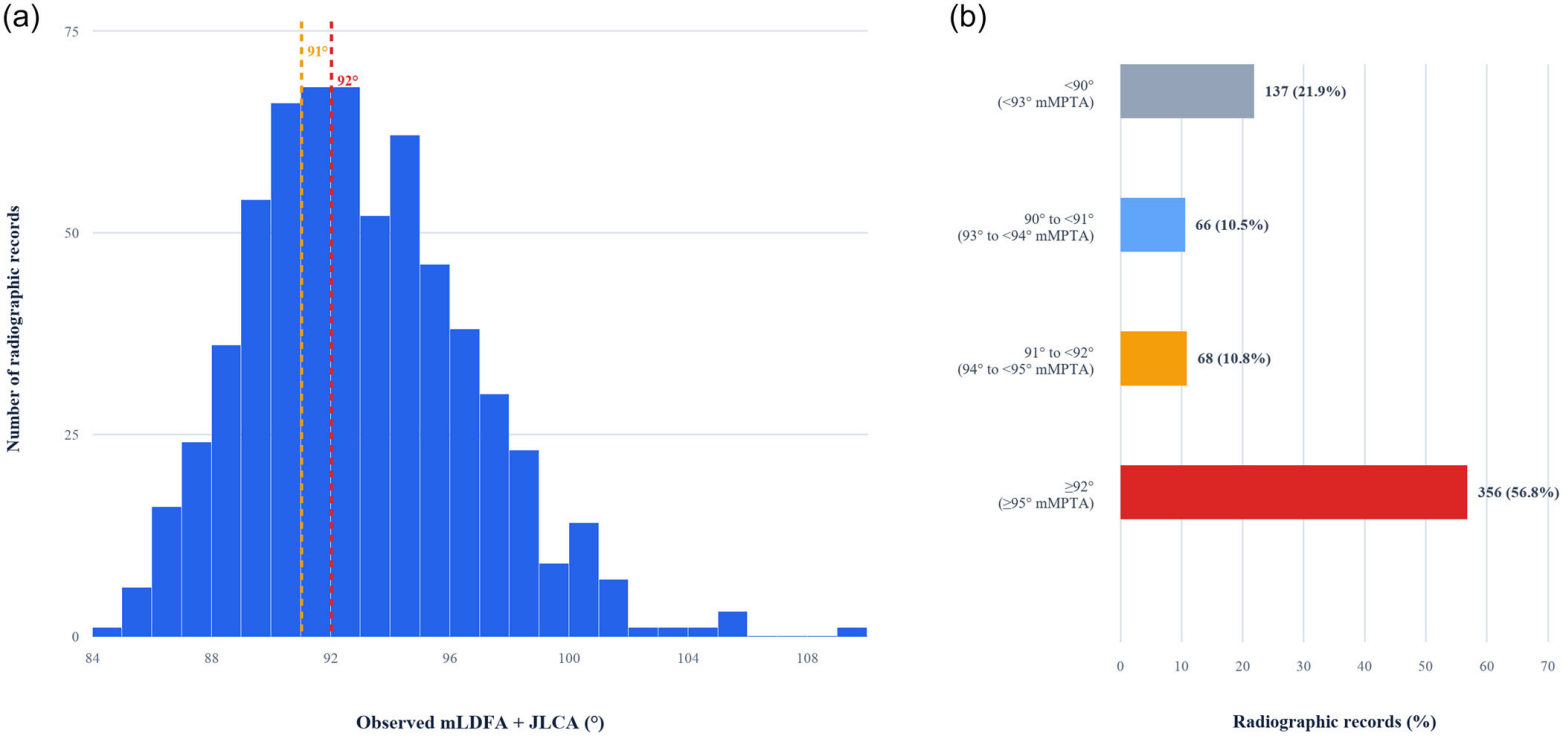
Observed distribution of mechanical lateral distal femoral angle plus joint line convergence angle. (a) Observed distribution of mechanical lateral distal femoral angle (mLDFA) plus joint line convergence angle (JLCA). (b) Proportions of radiographic records across the prespecified mLDFA + JLCA intervals: <90°, 90° to <91°, 91° to <92°, and ≥92°.

### Supplementary and patient‐level descriptive analyses

Supporting Information: Tables S1–S5 report the Chinese‐threshold source distribution, the one‐record‐per‐patient analysis, the full QC rules, supplementary target scenarios and K‐L grading reliability.

The supplementary analysis retained the lowest‐HKA eligible record for each of 392 patients. At the 3° valgus target, mean calculated mMPTA was 96.5 ± 3.9°. A value of at least 94° was found in 290/392 (74.0%; 95% CI, 69.4%–78.1%), and a value above 95° was found in 245/392 (62.5%; 95% CI, 57.6%–67.1%). For K‐L grading, first‐session interrater weighted kappa showed poor agreement (0.314), compared with 0.667 in the second session after the general criteria review. Corresponding ICC(A,1) values were 0.320 and 0.672. Intrarater weighted kappa was 0.800 for reader 1 and 0.844 for reader 2, with corresponding ICC(A,1) values of 0.804 and 0.847 (Supporting Information: Table S5).

## DISCUSSION

At the primary HKA target of 3° valgus, mean calculated mMPTA was 96.0 ± 3.7°, and 424/627 (67.6%; 95% CI, 63.9%–71.2%) reached the prespecified 94° planning‐review threshold. The most frequent deformity‐source patterns were intraarticular alone (24.7%) and tibial‐side plus intraarticular (24.4%). Under the fixed‐mLDFA and fixed‐JLCA assumptions, assigning the full correction to the tibia, therefore, often implied a high mMPTA. The observed proportion quantifies a familiar concern in OWHTO planning: achieving the whole‐limb target can require substantial alteration of local tibial orientation. HKA and weight‐bearing‐line targets describe the intended whole‐limb alignment [7, 10, 15, 22], while the ESSKA consensus and joint‐line studies recommend reviewing mMPTA and JLO during tibial correction [5, 24].

At a fixed HKA target, calculated mMPTA is the sum of the target valgus angle, mLDFA and JLCA. The latter two variables are components of the calculation rather than independently estimated risk factors. Each supplementary target translates the same measured distribution, so the scenarios describe geometric consequences rather than independent clinical comparisons. At targets closer to neutral, fewer records had a calculated mMPTA of at least 94°: 45.9% at 1° and 56.8% at 2°, compared with 67.6% at the primary 3° target. These arithmetic differences do not show that a lower target is clinically superior. Target selection still depends on compartment disease, cartilage status, symptoms, weight‐bearing‐line goals and the full correction strategy [5, 7, 10, 15, 22].

The source‐pattern analysis helps explain why some records had a high calculated mMPTA. When femoral or intraarticular varus contributes to whole‐limb alignment, assigning the full correction to the tibia transfers those components into the calculated mMPTA. This interpretation supports systematic assessment of deformity source during planning [3]. Studies of double‐level osteotomy provide context for selected complex deformities [1, 2]; the present geometric analysis does not compare clinical outcomes or establish indications for a particular procedure. In a North American operative cohort, isolated tibial varus was the most frequent of four bony deformity categories [12]. That younger, surgically selected population and its bony classification differ from the present symptomatic radiographic sample. The proportion classified as having no threshold‐defined abnormality changed from 22/627 (3.5%) with conventional Paley‐style ranges to 233/627 (37.2%) with Chinese sex‐specific reference limits. Deformity‐source categories are therefore sensitive to the reference population and threshold definitions; this label does not establish anatomical normality or suitability for surgery. The continuous mMPTA calculation does not use these category boundaries. JLCA has also been associated with medial KOA severity, meniscal extrusion and cartilage lesions [29], supporting its interpretation beyond bony shape alone.

JLCA represents an intraarticular contribution to coronal alignment that can change with loading and after OWHTO. JLCA‐aware planning has improved radiographic correction accuracy by allowing for this contribution [11, 18]. If bony correction is planned from a standing radiograph without allowing for a subsequent JLCA decrease, the achieved HKA may become more valgus than intended. For the same target HKA and fixed mLDFA, each 1° of expected JLCA reduction decreases the required bony correction and calculated mMPTA by 1°. The fixed‐JLCA calculation therefore describes a specified planning scenario rather than an unconditional upper bound on postoperative geometry. The practical role of JLCA assessment is to distinguish the correction expected from a reducible intraarticular component from that requiring bone correction. In a retrospective study targeting 5° valgus, JLCA‐adjusted planning reduced correction outliers greater than 2° from 24% to 9% [18]. Standing radiographs describe alignment under load, while supine and valgus‐stress views can help assess correctability. This assessment can inform the expected JLCA change and a corresponding reduction in planned bony correction. The required tibial correction can then be recalculated, and the resulting mMPTA and joint‐line orientation reviewed. Valgus‐stress views provide complementary information, but stress‐based simulation alone may overestimate the postoperative weight‐bearing‐line ratio [8]. Among knees with preoperative JLCA > 2° in an inverted V‐shaped HTO cohort, postoperative JLCA was associated with both preoperative JLCA and postoperative HKA [26], illustrating its dependence on achieved alignment.

The 94° threshold is a trigger for planning review. The mMPTA measures proximal tibial joint‐line orientation relative to the tibial mechanical axis, whereas weight‐bearing JLO also depends on whole‐limb alignment and stance. These measures are related but not interchangeable. In a postoperative cohort, mMPTA of 95° or greater did not necessarily produce JLO of 5° or greater [13]. Clinical follow‐up studies have not found uniformly poorer outcomes with increased postoperative mMPTA; [9, 31] one reported no significant deterioration of minimum 10‐year outcomes with mMPTA greater than 95° [31]. Thus, the calculated 94° threshold identifies plans for closer assessment of joint‐line geometry, not a validated clinical failure cutoff. Patient‐specific finite‐element studies provide a research framework for examining the biomechanical consequences of HTO constructs [20]. Integrating bone geometry, loading and soft‐tissue response in individualised digital‐twin planning remains a future direction requiring independent validation against postoperative measurements and clinical outcomes.

## LIMITATIONS

This was a single‐centre retrospective radiographic study without postoperative mMPTA, correction accuracy, patient‐reported outcomes or long‐term survival. The source radiographs were obtained for selected clinical indications, so the cohort does not represent all patients attending the clinic or a selected OWHTO candidate population. The calculation held mLDFA and JLCA constant, although JLCA can change after OWHTO. Because some patients contributed bilateral or repeated records, the primary dispersion estimates may underestimate between‐patient variability. The Wilson CIs do not account for this correlation and may be too narrow. Retaining one record per patient provided a descriptive sensitivity analysis with different selection and weighting. Body mass index was unavailable. K‐L reliability was assessed in 40 patients, and first‐session interrater agreement was poor. General criteria review between sessions means that the two‐session results do not measure repeatability under unchanged reading conditions. Prospective studies are needed to assess the clinical relevance of the 94° review threshold and the reproducibility of deformity‐source classifications across populations.

## CONCLUSIONS

At a 3° valgus target, tibia‐only planning implied an mMPTA of at least 94° in about two‐thirds (67.6%) of records from this varus knee osteoarthritis cohort. Preoperative planning should therefore assess local tibial joint‐line orientation and deformity source alongside the whole‐limb alignment target.

## AUTHOR CONTRIBUTIONS

**Xu Jiang**: Conceptualisation; data curation; formal analysis; methodology; writing—original draft; writing—review and editing. **Yicang Wang**: Formal analysis; writing—original draft; writing—review and editing. **Xing Li**: Conceptualisation; methodology; writing—original draft; writing—review and editing. **Songtao Ai**: Data curation; writing—review and editing. **Jun Zhou**: Methodology; writing—review and editing. **Kai Xie**: Data curation; supervision; writing—review and editing. **Liao Wang**: Conceptualisation; supervision; writing—review and editing. **Mengning Yan**: Conceptualisation; supervision; writing—review and editing.

## FUNDING INFORMATION

This work was supported by the Interdisciplinary Program of Shanghai Jiao Tong University (‘Med‐X Star’ Program) funded by the Fundamental Research Funds for the Central Universities (Grant Nos. YG2025LC12 to L.W. and YG2025QNA04 to X.J.), the Science and Technology Commission of Shanghai Municipality (Grant No. 23S31905900 to L.W.), the ‘Jiu Lian’ Joint Research Project of Shanghai Ninth People's Hospital, Shanghai Jiao Tong University School of Medicine (Grant No. JL202605 to L.W.), the Shanghai Municipal Talents Work Bureau (Grant No. QNDS2025020 to K.X.) and the National Natural Science Foundation of China (Grant Nos. 82202680 to K.X. and 12272232 to M.Y.).

### USE OF GENERATIVE AI AND AI‐ASSISTED TECHNOLOGIES

GPT‐6 was used to assist with language editing, calculation‐code preparation and figure preparation. This assistance was separate from the Skyward system used for radiographic measurement. The authors retain responsibility for the scientific content and final decisions.

## CONFLICT OF INTEREST STATEMENT

Author Xing Li is an employee of Yingwei Medical Technology (Shanghai) Co. Ltd., the developer of Skyward, the AI‐assisted radiographic measurement software used in this study. To mitigate potential bias, study eligibility decisions, quality‐control adjudication, statistical analyses, interpretation of clinical findings and final conclusions were independently performed or verified by authors with no financial relationship with the company. The remaining authors declare no competing interests.

## ETHICS STATEMENT

This retrospective radiographic geometry study was conducted in accordance with the Declaration of Helsinki and was approved by the Medical Ethics Committee of Shanghai Ninth People's Hospital, Shanghai Jiao Tong University School of Medicine (approval no. SH9H‐2023‐T97‐1). The requirement for written informed consent was waived because all data were deidentified before analysis.

## REPORTING GUIDELINE

The manuscript was prepared with reference to STROBE principles for observational studies and with explicit limitations for retrospective radiographic geometry research.

## Supporting information

Supporting File 1.

## Data Availability

The source radiographs and raw clinical database are not publicly available because they contain sensitive medical information. Deidentified analysis data and analysis code may be made available from the corresponding author upon reasonable request, subject to institutional approval and applicable privacy regulations.
