# Supplementary material for: Tibia‐only planning to 3° valgus implies mechanical medial proximal tibial angle ≥94° in about two‐thirds of varus knee osteoarthritis radiographs
Source: J Exp Orthop. 2026 Nov 4;13(4):e70977. doi: 10.1002/jeo2.70977 (PMC13733366; doi:10.1002/jeo2.70977)
Supplement: Supplementary file 1 [file JEO2-13-e70977-s001.docx]

**Supplementary Material**

Supplementary material for: Tibia-only planning to 3° valgus implies mMPTA ≥94° in about two-thirds of varus knee osteoarthritis radiographs

**Supplementary Table 1. Deformity-source patterns under Chinese sex-specific ±2 standard deviation thresholds.**

| **Deformity-source pattern** | **Records** | **Proportion** |
| --- | --- | --- |
| Intra-articular | 139 | 22.2% |
| Tibial-side + intra-articular | 56 | 8.9% |
| Tibial-side | 153 | 24.4% |
| Femoral-side + intra-articular | 11 | 1.8% |
| Tibial-side + femoral-side + intra-articular | 3 | 0.5% |
| Tibial-side + femoral-side | 10 | 1.6% |
| Femoral-side | 22 | 3.5% |
| No threshold-defined abnormality | 233 | 37.2% |

Note. Chinese sex-specific thresholds were derived from the mean ±2 SD reference limits in a non-KOA Chinese population. Records and proportions are reported as n and %, respectively. Percentages may not total 100% because of rounding. KOA, knee osteoarthritis; SD, standard deviation.

**Supplementary Table 2. Patient-level one-knee descriptive sensitivity analysis.**

| **Analysis** | **Result** |
| --- | --- |
| Cohort | 392 records, retaining one eligible record per patient |
| Selection rule | Record with the lower HKA when more than one record was eligible |
| Calculated mMPTA at 3° valgus | 96.5 ± 3.9° |
| mMPTA >=94° at 3° valgus | 290/392 (74.0%; 95% CI, 69.4 to 78.1%) |
| mMPTA >95° at 3° valgus | 245/392 (62.5%; 95% CI, 57.6 to 67.1%) |
| Interpretation | Descriptive check of unit-of-analysis choice; not an independent validation of the deterministic calculation |

Note. One eligible radiographic record was retained per patient; when more than one record was eligible, the record with the lower HKA was selected. Values are reported as mean ± SD or n (%; 95% Wilson CI). This selection changes the weighting and is a descriptive unit-of-analysis check. CI, confidence interval; HKA, hip-knee-ankle angle; mMPTA, mechanical medial proximal tibial angle; SD, standard deviation.

**Supplementary Table 3. Prespecified quality-control rules and planning-calculation assumptions.**

| **Item** | **Prespecified rule or assumption** | **Observed result** |
| --- | --- | --- |
| Source export | Knee-side radiographic records | 1426 records |
| KOA records | Database label | 979 records |
| Age extraction | First integer from age field; 18 to 100 years | 1426/1426 usable |
| Angular range check | mLDFA 75-105; mMPTA 70-105; JLCA -15 to 15; HKA 140-220° | 1420/1426 passed |
| Formula check | Absolute residual <=0.01° for HKA = mMPTA + (180 - mLDFA) - JLCA | 1423/1426 passed |
| QC-passed KOA records | Range and formula check pass | 972 records |
| Main cohort | QC-passed KOA records with HKA <176.5° | 627 records from 392 patients |
| Planning calculation | mMPTA calculated at V° valgus = V + mLDFA + JLCA; mLDFA and JLCA held constant | Deterministic calculation, not a measured postoperative outcome |
| Source records | Raw export preserved | No source values overwritten |

Note. HKA, hip-knee-ankle angle; JLCA, joint line convergence angle; KOA, knee osteoarthritis; mLDFA, mechanical lateral distal femoral angle; mMPTA, mechanical medial proximal tibial angle; QC, quality control.

**Supplementary Table 4. Calculated mMPTA under supplementary HKA target scenarios.**

| **Target valgus** | **Target HKA** | **Calculated mMPTA, mean ± SD** | **mMPTA ≥94°, n (%; 95% CI)** | **mMPTA >95°, n (%; 95% CI)** |
| --- | --- | --- | --- | --- |
| 1° valgus | 181° | 94.0 ± 3.7° | 288/627 (45.9%; 95% CI, 42.1 to 49.8%) | 236/627 (37.6%; 95% CI, 33.9 to 41.5%) |
| 2° valgus | 182° | 95.0 ± 3.7° | 356/627 (56.8%; 95% CI, 52.9 to 60.6%) | 288/627 (45.9%; 95% CI, 42.1 to 49.8%) |
| 4° valgus | 184° | 97.0 ± 3.7° | 490/627 (78.1%; 95% CI, 74.7 to 81.2%) | 424/627 (67.6%; 95% CI, 63.9 to 71.2%) |
| 5° valgus | 185° | 98.0 ± 3.7° | 544/627 (86.8%; 95% CI, 83.9 to 89.2%) | 490/627 (78.1%; 95% CI, 74.7 to 81.2%) |
| 6° valgus | 186° | 99.0 ± 3.7° | 580/627 (92.5%; 95% CI, 90.2 to 94.3%) | 544/627 (86.8%; 95% CI, 83.9 to 89.2%) |

Note. Values are reported as mean ± SD or n (%; 95% Wilson CI). These record-level binomial intervals do not account for within-patient correlation and may be too narrow. The supplementary targets translate the same measured mLDFA + JLCA distribution; they are not clinical comparisons between target strategies. CI, confidence interval; HKA, hip-knee-ankle angle; JLCA, joint line convergence angle; mLDFA, mechanical lateral distal femoral angle; mMPTA, mechanical medial proximal tibial angle; SD, standard deviation.

**Supplementary Table 5. Reproducibility of Kellgren-Lawrence grading in 40 randomly selected patients.**

| **Comparison** | **Exact agreement** | **Within-one-grade agreement** | **Weighted kappa** | **95% CI** | **ICC(A,1), 95% CI** |
| --- | --- | --- | --- | --- | --- |
| Inter-rater, round 1 | 60.0% | 97.5% | 0.314 | 0.032 to 0.571 | 0.320 (0.033 to 0.577) |
| Inter-rater, round 2 | 80.0% | 100.0% | 0.667 | 0.458 to 0.846 | 0.672 (0.465 to 0.849) |
| Intra-rater, reader 1 | 90.0% | 100.0% | 0.800 | 0.596 to 0.950 | 0.804 (0.602 to 0.951) |
| Intra-rater, reader 2 | 87.5% | 100.0% | 0.844 | 0.690 to 0.961 | 0.847 (0.695 to 0.962) |

Note. Two orthopaedic surgeons independently graded one randomly selected knee from each of 40 patients on two occasions separated by 2 weeks. General K-L criteria were reviewed before session 2; study cases were not discussed and previous scores were not consulted. Readers remained blinded to each other's and their own previous ratings. Session-1 inter-rater agreement was poor; comparison across sessions includes the intervening criteria review and does not represent repeatability under unchanged conditions. Weighted kappa is quadratic-weighted Cohen kappa. ICC(A,1) is a two-way random-effects, absolute-agreement, single-measure intraclass correlation coefficient. CI, confidence interval; ICC, intraclass correlation coefficient; K-L, Kellgren-Lawrence.
